# Establishment and application of a CRISPR–Cas12a assisted genome-editing system in *Zymomonas mobilis*

**DOI:** 10.1186/s12934-019-1219-5

**Published:** 2019-10-03

**Authors:** Wei Shen, Jun Zhang, Binan Geng, Mengyue Qiu, Mimi Hu, Qing Yang, Weiwei Bao, Yubei Xiao, Yanli Zheng, Wenfang Peng, Guimin Zhang, Lixin Ma, Shihui Yang

**Affiliations:** State Key Laboratory of Biocatalysis and Enzyme Engineering, Environmental Microbial Technology Center of Hubei Province and School of Life Sciences, Hubei University, Wuhan, 430062 China

**Keywords:** *Zymomonas mobilis*, CRISPR, Cas12a, ssDNA recombineering, In situ mutagenesis, Lactate, Genome engineering

## Abstract

**Background:**

Efficient and convenient genome-editing toolkits can expedite genomic research and strain improvement for desirable phenotypes. *Zymomonas mobilis* is a highly efficient ethanol-producing bacterium with a small genome size and desirable industrial characteristics, which makes it a promising chassis for biorefinery and synthetic biology studies. While classical techniques for genetic manipulation are available for *Z. mobilis*, efficient genetic engineering toolkits enabling rapidly systematic and high-throughput genome editing in *Z. mobilis* are still lacking.

**Results:**

Using Cas12a (Cpf1) from *Francisella novicida*, a recombinant strain with inducible *cas12a* expression for genome editing was constructed in *Z. mobilis* ZM4, which can be used to mediate RNA-guided DNA cleavage at targeted genomic loci. gRNAs were then designed targeting the replicons of native plasmids of ZM4 with about 100% curing efficiency for three native plasmids. In addition, CRISPR–Cas12a recombineering was used to promote gene deletion and insertion in one step efficiently and precisely with efficiency up to 90%. Combined with single-stranded DNA (ssDNA), CRISPR–Cas12a system was also applied to introduce minor nucleotide modification precisely into the genome with high fidelity. Furthermore, the CRISPR–Cas12a system was employed to introduce a heterologous lactate dehydrogenase into *Z. mobilis* with a recombinant lactate-producing strain constructed.

**Conclusions:**

This study applied CRISPR–Cas12a in *Z. mobilis* and established a genome editing tool for efficient and convenient genome engineering in *Z. mobilis* including plasmid curing, gene deletion and insertion, as well as nucleotide substitution, which can also be employed for metabolic engineering to help divert the carbon flux from ethanol production to other products such as lactate demonstrated in this work. The CRISPR–Cas12a system established in this study thus provides a versatile and powerful genome-editing tool in *Z. mobilis* for functional genomic research, strain improvement, as well as synthetic microbial chassis development for economic biochemical production.

## Introduction

*Zymomonas mobilis* is a facultative anaerobic Gram-negative ethanologenic bacterium and generally regarded as safe (GRAS), which anaerobically ferments glucose, fructose and sucrose for ethanol production through Entner–Doudoroff (ED) pathway with many desirable characteristics, such as high specific glucose uptake rate, rapid catabolism, high tolerance of ethanol concentration up to 16% (v/v) and broad pH range (4–7.5) [1–4]. The metabolically engineered recombinant *Z. mobilis* broadens the fermentable substrates to pentose sugars such as xylose and arabinose, which is a promising microorganism for economic biochemical production from lignocellulosic biomass [5–7]. Moreover, many systems biology studies of *Z. mobilis* have been carried out with significant omics datasets accumulated, providing valuable information for strain improvement [8–14].

The capability of systematic and high-throughput modifying microbial genome for desirable phenotypes represents a great advancement for fully understanding of gene function and regulatory networks [15]. Conventional genetic studies in *Z. mobilis* are usually based on homologous recombination (HR) using suicide or unstable replicative vectors. Currently, metabolic engineering and synthetic biology methods have been improved rapidly and various genetic engineering tools including suicide plasmid-based mutant construction, site-specific flippase (FLP) recombination and transposon mutagenesis, as well as RecET recombination system have been applied in *Z. mobilis* for gene function analysis and metabolic engineering [7, 16, 17]. Among these methods, the RecET recombination system derived from bacteriophage λ-Red system provides an efficient tool to induce homologous recombination between linear DNA fragments and bacterial chromosomes with the use of selectable markers. However, inevitable obstacles exist with respect to the capability of these traditional methods. For example, the allelic exchange methods involve either introducing a selectable marker into the edited locus or a counter-selection system such as SacB, which generally are low efficiency, time-consuming, and laborious [18]. Moreover, these systems are usually restricted by the available selection markers and the expression of the recombinase in the host [19, 20]. Therefore, it is crucial to develop an efficient and convenient genetic engineering tool to expedite the strain development in *Z. mobilis*.

The Clustered Regularly Interspaced Short Palindromic Repeats (CRISPR)-CRISPR-associated (CRISPR–Cas) system is a widely distributed prokaryotic adaptive immunity system that confers resistance to invading genetic elements in archaea and bacteria [21–23]. CRISPR–Cas systems consist of CRISPR arrays and Cas proteins. Each CRISPR RNA (crRNA) produced from CRISPR transcripts is complementary to a target sequence and thus guides the Cas proteins to recognize and cleave the target in a sequence-specific manner [24]. Currently, CRISPR–Cas systems have been grouped into seven main types (types I–VI plus type U) based on the system complexity and signature proteins, that are classified into two classes (class 1 and class 2) [24–26]. Class 1 systems (containing types I, III, and IV) typically form multi-subunit protein-crRNA complex, whereas class 2 systems (containing types II, V, and VI) use a single crRNA-guided protein for target interference [18, 26]. Functional CRISPR–Cas enzymes will introduce DNA injuries, *e.g.* double-stranded DNA breaks (DSB), within target sequences defined by guide RNAs (gRNA) [21, 23]. The resulting injuries will activate endogenous DNA repair mechanisms, such as nonhomologous end-joining (NHEJ) and homologous recombination (HR) [27, 28]. As most bacteria including *Z. mobilis* lack the efficient but error-prone NHEJ pathway, they repair DNA injuries rely largely on the HR in combination with donor templates. DNA repair through HR would give precise edits.

The type II CRISPR–Cas9 system from *Streptococcus pyogenes* has been exploited as a highly efficient genetic tool in various organisms for gene editing and regulation due to its simplicity and versatility [20, 29, 30]. However, the potential toxicity of Cas9 nuclease limited its broad application in certain prokaryotic hosts such as *Corynebacterium glutamicum* and *Cyanobacteria* sp. [31, 32]. This would explain, at least partly, the fact that although this system has been employed for the native plasmid curing in *Z. mobilis* [33], no follow-up report on its application for genome engineering is accumulated in the literature.

Recently, Cas12a (Cpf1), a Type V RNA-programmable endonuclease, has also been characterized and engineered for genome editing [30, 34, 35], which was reported to have less toxicity to the prokaryotic cells compared to Cas9 [24, 31, 32]. Cas12a guided by a mature crRNA recognizes a protospacer flanked by a T-rich PAM (protospacer-adjacent motif) and creates staggered ends while Cas9 typically uses a G-rich PAM sequence for target discrimination and generates blunt ends [34, 36–40]. Collectively, Cas12a would be taken as an alternative or complement to Cas9 for prokaryotic engineering. Zetsche et al., applied in vitro PAM identification assay and confirmed the PAM for Cas12a as 5′-TTN [34]. Leenay et al. performed a comprehensive screening of PAM with the catalytically dead Cas12a indicated that PAM sequences of GTTC and TTTN yielded stronger repression in comparison to a non-targeting RNA control [37].

In this study, we aim to explore the possibility to develop a highly efficient, convenient genetic engineering tool for *Z. mobilis* using the CRISPR–Cas12a system to facilitate our understanding of this ethanologic bacterium and to expedite the practices on metabolic engineering and genome engineering.

## Results and discussions

### Construction and functionality of genomic integrated Cas12a in *Z. mobilis* ZM4

**Fig. 1:**
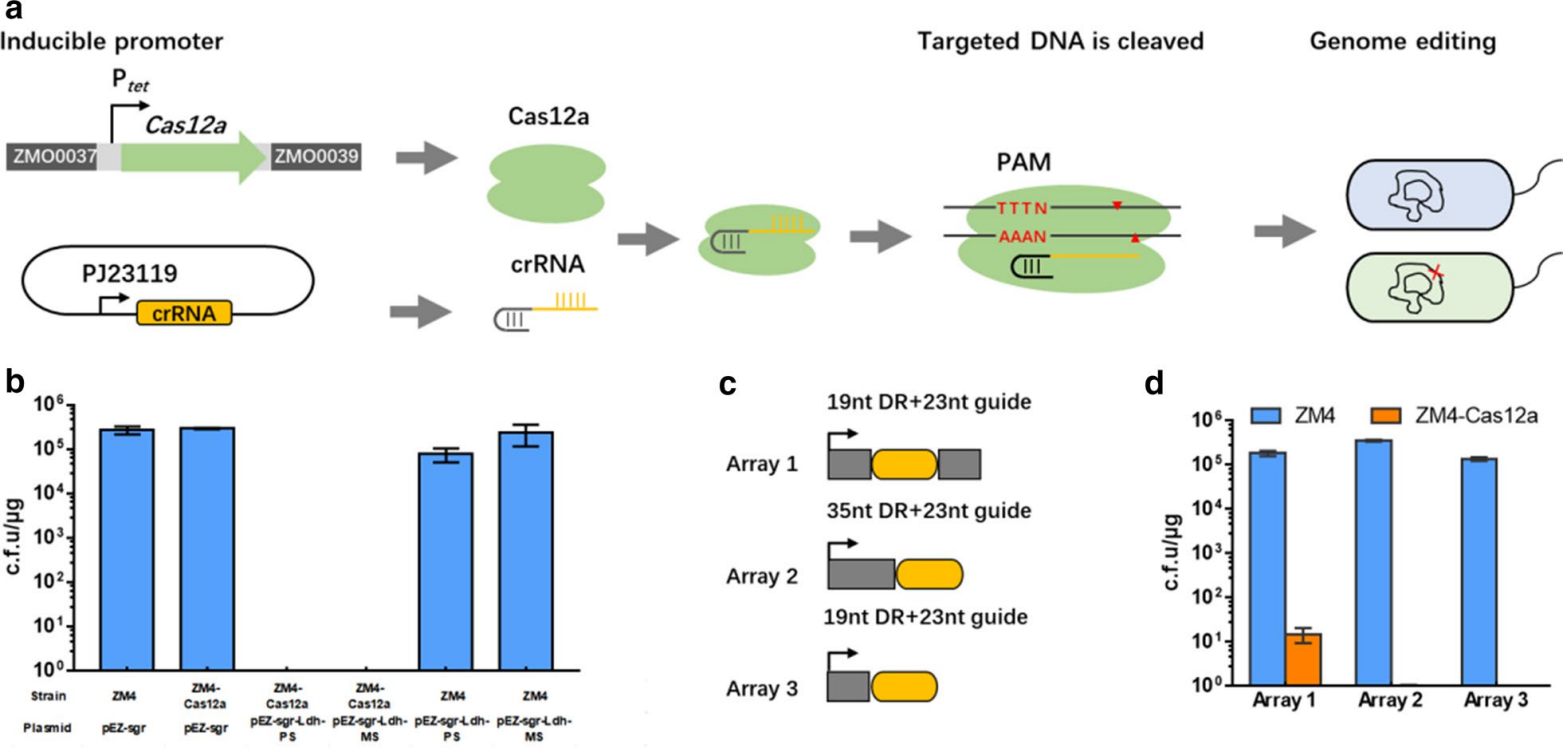
Scheme and genome editing using the heterologous CRISPR–Cas12a system. Scheme of genome editing with Cas12a using a single plasmid approach. Tetracycline-inducible Cas12a is directed to specific DNA targets by constitutively expressed sgRNAs. Cas12a was stably integrated into the *ZMO0038* locus, and the assistant plasmid constitutively express sgRNAs (**a**). Colony forming unit (c.f.u) of *Z. mobilis* ZM4 and the Cas12a expressing strain with or without the expression of crRNA targets (**b**). Genome editing mediated by Cas12a with different versions of artificial CRISPR arrays. Array 1 crRNAs are in a double direct repeat form (19-nt DR with 23-nt guide); Array 2 crRNAs are in a single direct repeat form (35-nt DR with 19-nt guide); and Array 3 crRNAs are in their mature form (19-nt DR with 23-nt guide) (**c**). c.f.u of *Z. mobilis* expressing the nuclease with different versions of artificial CRISPR arrays (**d**). Values are the means of experiments with three or more technical replicates; error bars are standard deviations

We initially attempted to establish the CRISPR–Cas12a-based toolkit by expressing both the Cas12a effector nuclease and the crRNA via a single plasmid. However, transformation with this plasmid could only generate few transformants with a very poor transformation efficiency (Additional file 1: Fig. S1), possibly due to its relatively large size and plasmid copy number effect, hence leading to the failure in genome editing. To address this issue, a recombinant strain (ZM4-Cas12a) was constructed by integrating a *cas12a*-expressing cassette driven by the tetracycline-inducible promoter *Ptet*, together with a spectinomycin antibiotic marker for selection, into the *ZMO0038* locus through homologous recombination (Fig. 1a).

In order to assist CRISPR–Cas12a-based genome editing in *Z. mobilis*, self-targeting plasmids were constructed to individually express crRNAs consisting of a 19-nt direct repeat (DR) and a 23-nt guide sequence. It was reported that this crRNA species exhibited optimal performance for genome editing [34]. Following this principle, two plasmids, pEZ15a-sgr-PS-Ldh and pEZ15a-sgr-MS-Ldh, were designed to respectively target a sequence on the coding or noncoding strand of a lactate dehydrogenase encoding gene *ZMO1237* (*ldh*).

We next assayed the DNA cleavage activity of the exogenously introduced CRISPR–Cas12a system. To this end, the self-targeting plasmids were individually introduced into either ZM4 or ZM4-Cas12a cells. Notably, whereas transformation of ZM4 cells yielded a very high transformation efficiency reaching 10^5^ c.f.u/μg DNA, transformation of ZM4-Cas12a cells got a thousand folds lowered transformation efficiency (Fig. 1b). By contrast, transformation of the crRNA-expressing plasmid into ZM4 cells or the plasmid vector (pEZ-sgr) into ZM4-Cas12a obtained high efficiency. Interestingly, even in the absence of tetracycline, expression of crRNA targeting the *ldh* gene in *Z. mobilis* still conferred a nearly 100% killing frequency, indicating a basal expression of Cas12a driven by *Ptet* at the condition without tetracycline induction as reported before [41]. Collectively, these results suggested that the CRISPR–Cas12a system can efficiently mediate targeted DNA cleavage, and the Cas12a expression alone in *Z. mobilis* is not toxic.

To further develop a compatible multiplex gene-editing strategy in *Z. mobilis*, we evaluated effects of crRNA variants on directing self-killing. The crRNAs variants all contain a 23-nt guide sequence targeting the *ldh* locus of *Z. mobilis* genome but differed in DRs: in Array 1, the guide is sandwiched by two 19-nt DRs while in Array 2 and 3 only a downstream 35-nt or 19-nt repeat, respectively, is present (Fig. 1c). All the crRNAs efficiently directed the nuclease activity of the chromosomally integrated Cas12a to the target sequence for destruction, as almost no transformant could be obtained when the constructs expressing the crRNA variants were introduced into ZM4-Cas12a cells via electroporation (Fig. 1d). Few transformants were obtained from transformation with the Array 1-containing plasmid, which were subsequently identified to be escapers carrying a mutated Array 1. Sanger sequencing results revealed that recombination occurred between the two 19-nt repeats, leading to the loss of the guide sequence.

### Application of CRISPR–Cas12a system for native plasmid curing in *Z. mobilis*

Recently, a revised *Z. mobilis* ZM4 genome sequence (~ 2.06 Mb) was released, which includes four native plasmids named pZM32 (32,791 bp), pZM33 (33,006 bp), pZM36 (36,494 bp), and pZM39 (39,266 bp) according to their sequence sizes [9]. Sequencing analysis revealed that all four plasmids encode replicases that are required for their replication. Thus, if these replicase genes were inactivated using the CRISPR–Cas12a-based toolkit, the plasmid would lose the ability for replication and will then be consequently cured [9, 33].

**Fig. 2:**
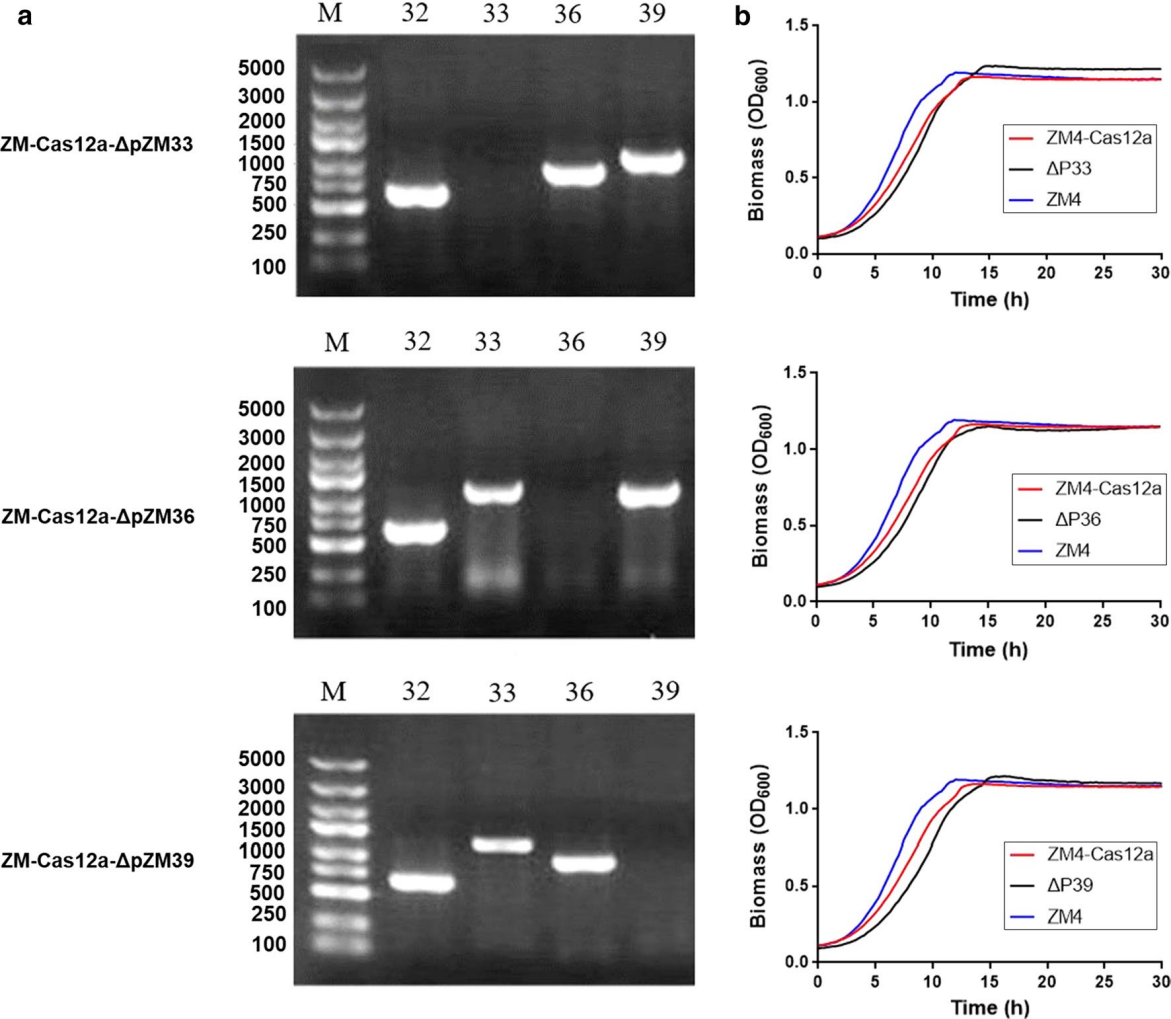
Native plasmid curing in *Z. mobilis* by CRISPR–Cas12a system and the impact of plasmid curing on cellular growth. The native plasmid curing was identified by colony PCR. Primers specific for native plasmids of pZM33 (P33-Check-F/R, 1192 bp), pZM36 (P36-Check-F/R, 896 bp) and pZM39 (P39-Check-F/R, 1121 bp) were used to verify the existence of each plasmid, respectively (**a**). The growth curve of *Z. mobilis* and plasmid-deleted mutants using Bioscreen C (**b**)

Genes encode replicases for the native plasmids of pZM33, pZM36, and pZM39 are pZM33_028 (NZ_CP023679), pZM36_036 (CP023680), and pZM39_032 (CP023681) respectively. gRNAs being designed to specifically target sequences within the replicase-encoding genes were expressed from the plasmids pEZ15a-sgr-ΔpZM32, pEZ15a-sgr-ΔpZM33, pEZ15a-sgr-ΔpZM36 and pEZ15a-sgr-ΔpZM39, respectively. A total of 200 ~ 300 ng of each plasmid DNA was transformed into ZM4-Cas12a. Primers specifically against each native plasmid were designed to further verify plasmid curing. Our results indicated that three native plasmids, pZM33, pZM36 and pZM39, were cured independently in one step with 100% efficiency (Fig. 2a), and the removal of these three native plasmids individually had no or negligible influence on cell growth (Fig. 2b).

Plasmids play pivotal role in the advancement of molecular biology, and various plasmid vectors for genetic and metabolic engineering purposes have been developed using an origin of replication region from native plasmids of *Z. mobilis* [42]. Eight *Z. mobilis* strains have been completely sequenced and contain 2–8 native plasmids with different sizes [9, 33]. Plasmid stability and compatibility issues will occur when an engineered plasmid with the same origin of replication is introduced into the host strain, which will limit the application of this powerful tool for strain improvement [43]. The CRISPR–Cas12a-based toolkit established here for *Z. mobilis* was applied for native plasmid curing successfully, which will facilitate the investigation of plasmid gene function and future genome reduction of *Z. mobilis*.

### Application of CRISPR–Cas12a assisted ssDNA recombineering for nucleotide substitutions in *Z. mobilis*

To introduce specific genomic changes, single strand oligonucleotide (ssDNA) with homology to the sequence flanking the DSB was used as templates to facilitate the repair of the damaged DNA. We tested this system by attempting to introduce point mutations into the *ldh* gene using ssDNA oligonucleotide recombination. Mutation of targeting genome was designed to alter its seed sequence with two mismatching nucleotides, which will also generate a *Pst*I restriction site. The recombinants with the engineered sequence can be checked by colony PCR and further verified by *Pst*I digestion, such that the recombination efficiency can be evaluated.

**Fig. 3:**
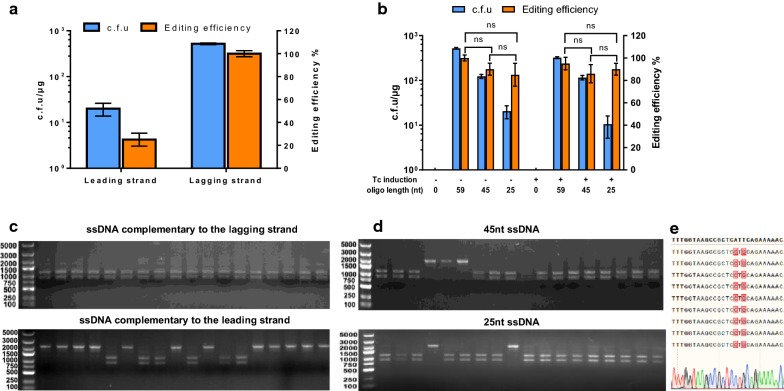
The impact of crRNA on lagging and leading strand as well as the length of direct repeats in artificial CRISPR arrays on transformation and editing efficiencies of the CRISPR–Cas12a assisted ssDNA recombineering in *Z. mobilis*. c.f.u and editing efficiency of *ldh* after editing by 59 nt template oligonucleotide targeting the lagging strand and leading strand, respectively (**a**), or by oligonucleotide template with different length targeting the lagging strand mediated by plasmid pEZ-sgr-ldh expressing *ldh* targeting crRNA (**b**). Transformants generated from CRISPR–Cas12a assisted ssDNA recombination targeting on lagging strand, leading strand (**c**), or oligonucleotide template with different length targeting the lagging strand (**d**). Transformants were screened by colony PCR with primer pair Ldh-check-F/R, and the PCR product was then digested with a *Pst*I to investigate the recombination efficiency. A 2.1 kb fragment indicates wild-type genotype, whereas the presence of 1.2 and 0.9 kb fragments indicate recombinant genotypes. The representative *ldh* recombinants were confirmed by Sanger sequencing (**e**). A multiple comparison between groups was performed for the editing efficiencies using different length of ssDNA through analysis of variance (ANOVA) followed by unpaired two-tailed student-*t* test using GraphPad InStat software (GraphPad, Prism 8). P value < 0.05 was considered to be statistically significant, and ns represents non-significant. Values are the means of experiment with three or more technical replicates, error bars are standard deviation

Targeting plasmid pEZ-sgr-Ldh-PS (~ 200 ng) was co-transformed with 1 μg ssDNA editing template. There was an apparent strand bias for the editing efficiency. Targeting the lagging strand showed almost 100% editing efficiency, which was four folds higher than that of targeting the other (Fig. 3a). This result is consistent with previous reports in *Mycobacterium smegmatis* and *C. glutamicum* [18, 31]. The length of editing template also affected the transformation efficiency with reduced colony forming unit (c.f.u), whereas it had litter effect on editing efficiency (Fig. 3b). These data indicated that the CRISPR–Cas12a-assisted ssDNA recombineering system can be served as a robust and precise tool for introducing minor nucleotide substitutions in *Z. mobilis*.

### Application of CRISPR–Cas12a system for gene deletion and replacement

**Fig. 4:**
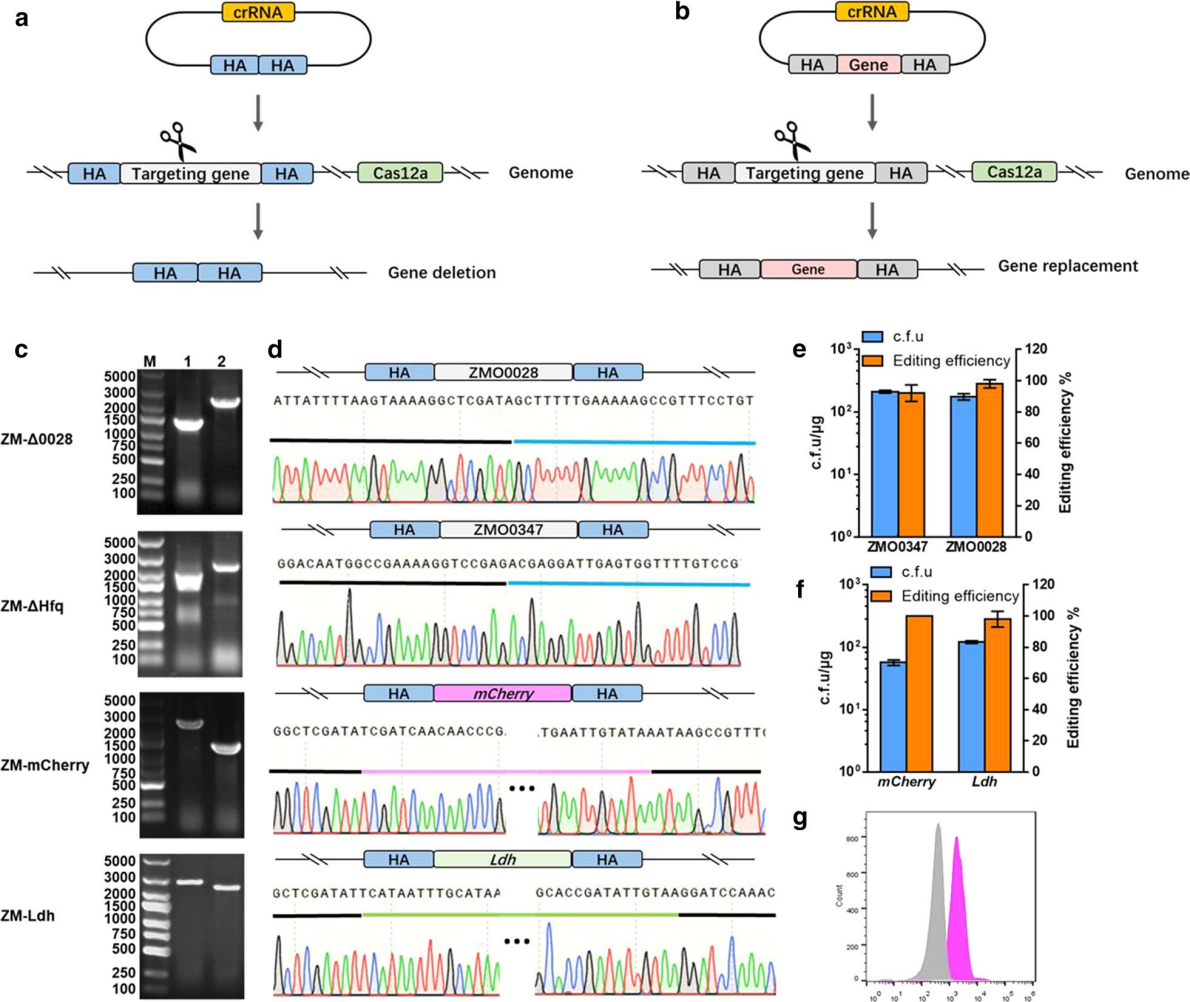
CRISPR-Ca12a mediated gene deletion and replacement in *Z. mobilis.* The scheme of CRISPR–Cas12a mediated gene deletion (**a**) and replacement (**b**). Co-expression of Cas12a and crRNA that targets the *Z. mobilis* chromosome introduced double strand break (DSB). Donor plasmid contains the desired edit as well as additional homologous sequence flanking upstream and downstream of the target locus. Transformants generated from CRISPR–Cas12a assisted genome recombination were verified by colony PCR. For deletion of *ZMO0028*, 2.3 kb PCR fragment generated by primer pair 0028check-F/R indicates the wild-type genotype, whereas the presence of 1.5 kb PCR fragment indicate the recombinant genotype. For deletion of *ZMO0347*, 2.8 kb PCR fragment generated by primer pair 0347check-F/R represents the wild-type genotype, whereas the presence of 2.1 kb PCR fragment indicates the recombinant genotype. For *mCherry* insertion, 1.5 kb PCR fragment generated by primer pair 0028check-F/R indicates ZMO0028 mutant genotype, whereas the presence of 2.5 kb PCR fragment indicates the recombinant genotype. The insertion of *LdhBc* was identified by primer pair 0028check-F/R. A 2.3 kb PCR fragment indicates the wild-type genotype, while a 2.9 kb PCR fragment indicates the insertion of *LdhBc*. M represents DNA marker (DL5,000), 1 and 2 represents PCR pduducts from recombiant and wild-type strains, respectively (**c**), and the representative recombinants were then confirmed by Sanger sequencing (**d**). c.f.u and editing efficiency of Cas12-assisted in-frame deletions (**e**), c.f.u and editing efficiency of Cas12-assisted *mCherry* and *LdhBc* insertion (**f**). A mutant with endogenous gene *ZMO0028* replaced by the reporter gene *mCherry* was also verified by flow cytometry, gray peak was signal from negative control ZM4 and pink peak from mutant ZM-mCherry (**g**). Values are the means of experiments with three or more technical replicates, error bars are standard deviation. *HA* homologous arm

Homologous recombination (HR) can be utilized for accurate sequence modifications such as nucleotide substitutions or gene insertion when a homologous DNA donor template is provided in conjunction with the generation of the DSB [19]. Genome editing can therefore be initiated by generating site-specific DSB in the genome. In order to utilize HR for gene editing, a DNA repair template containing the desired sequence must be delivered into cells of interest with the crRNA and Cas12a. The repair template contains the desired edit as well as additional homologous sequences flanking upstream and downstream of the target locus (Fig. 4a, b).

*ZMO0028* is a putative methylated adenine recognition and restriction gene belonging to the type IV R-M system, and the deficient of *ZMO0028* improved transformation efficiency of *Z. mobilis* [44]. *ZMO0347* encodes the RNA chaperone Hfq involving in tolerance against multiple hydrolysate inhibitors such as acetate, vanillin, furfural, and HMF in *Z. mobilis* [45–47]. Since both genes are not essential for cell viability, gRNAs were designed to target the *ZMO0028* and the *ZMO0347* loci for gene deletion using the CRISPR–Cas12a assisted genome-editing system developed in this work. A pair of homologous arms was selected from the flanking sequences of *ZMO0028* or *ZMO0347* and cloned into the pEZ-sgr vector, generating the donor-carrying plasmid. The deletion efficiency of *ZMO0028* or *ZMO0347* reached 90–100% (Fig. 4e). The obtained recombinants were further identified by PCR (Fig. 4c), and the PCR product was confirmed by Sanger sequencing (Fig. 4d). Those results indicated that HR-induced precise mutagenesis is particularly useful for generating markrless deletion mutations.

In the case of gene replacement, gRNA was designed for generating DSB within the *ZMO0028* locus. A reporter gene (*mCherry*) driving by a constitutive promoter *Pgap* and a lactate dehydrogenase-encoding gene *ldh* from *Bacillus coagulans* (*LdhBc*) under the control of the constitutive strong promoter *PadhB*, sandwiched by two recombination arms respectively homologous to the up- or down-flanking sequences of the *ZMO0028* locus, were cloned into the pEZ-sgr vector to generate the corresponding donor-carrying plasmids. Transformation with 1 μg of *mCherry* crRNA specific plasmid into *Z. mobilis* competent cells produced more than 40 c.f.u., among which 95% (Fig. 4f) were correctly edited (Fig. 4c, d). The edited strain with mCherry gene driven by *Pgap* promoter inserted into *ZMO0028* was then characterized by flow cytometry, and the fluorescence of recombinant strain showed a fluorescence shift compared with that of the wild-type strain (Fig. 4g).

### Characterization of a lactate-producing *Z. mobilis* strain constructed by the CRISPR–Cas12a system

**Fig. 5:**
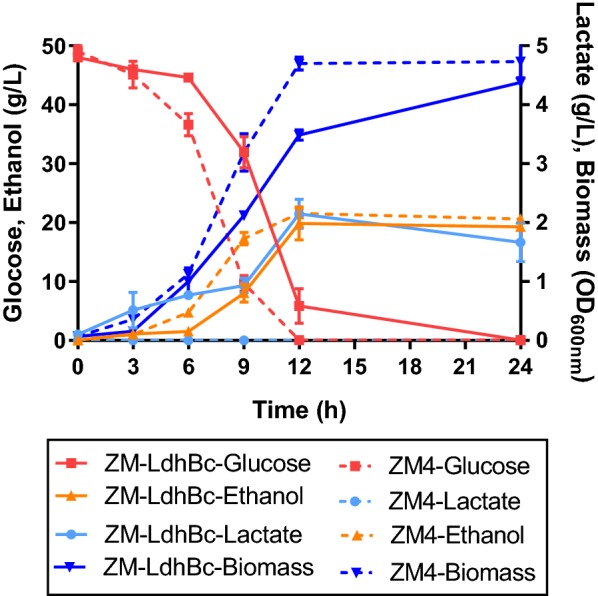
Growth curve, glucose consumption as well as ethanol and lactate production in ZM-LdhBc. The titer (g/L) of major end products detected in the supernatant of wild-type ZM4 and lactate producing *Z. mobilis* strains. Biomass was evaluated by determination of optical density (OD_600nm_). Values are the means of experiment with three or more technical replicates, error bars are standard deviation

Recombinant strain ZM-LdhBc harboring heterologous lactate gene developed in this study was then cultured in 100-mL shake flasks with 80 mL RMG5 medium containing 50 g/L glucose. The results exhibited that ZM-LdhBc had a significant improvement in lactate production with lactate titer reaching 2.12 g/L and a correspondingly reduced ethanol production from 21.25 to 18.31 g/L compared with the wild-type strain ZM4 (Fig. 5).

Those result thus demonstrated that the expression of a synthesized *LdhBc* under the control of the constitutive strong promoter *PadhB* is crucial for lactate production. However, the final ethanol titer was similar between the wild type and mutant strain, which indicated that *LdhBc* gene alone cannot effectively compete with ethanol production, and other strategy is needed to divert more carbon flux from ethanol production into lactate biosynthesis such as the knockout of pyruvate decarboxylase (PDC). However, PDC is essential for cell viability of the wild-type *Z. mobilis* and thus cannot be easily knocked out [48]. The high editing efficiency of CRISPR–Cas12a assisted ssDNA recombineering (ca. 100%) discussed above indicated that site-directed mutagenesis of *pdC* gene could be an alternative strategy to reduce the affinity of PDC to pyruvate and thus redirect the intermediate pyruvate for maximum lactate production.

## Conclusion

An efficient genome-editing tool based on the CRISPR–Cas12a was developed through the integration of Cas12a into the chromosome, and the impact of crRNA on lagging and leading strand as well as the length of direct repeats in artificial CRISPR arrays were further investigated to optimize the system, which was then successfully applied for plasmid curing, gene deletion and insertion as well as nucleotide substitution. In addition, the CRISPR–Cas12a system applied in this study for *Z. mobilis* was also used for metabolic engineering practices with a lactate-producing recombinant strain developed. The successful demonstration of the application of CRISPR–Cas12a system in *Z. mobilis* will extend the existing genetic toolbox for metabolic engineering and genome engineering in *Z. mobilis*, and can also be served as an example of developing CRISPR–Cas12a genome-editing tools in other microorganisms.

## Materials and methods

### Strains and culture conditions

*Escherichia coli* DH5α was used in this study for plasmid maintenance, which was cultured at 37 °C in Luria–Bertani medium (LB, 10 g/L tryptone, 5 g/L yeast extract, 10 g/L NaCl). *Z. mobilis* ZM4 was the parent strain for genetic modifications and was cultured at 30 °C in rich medium (RMG5: 10 g/L yeast extract, 50 g/L Glucose, 2 g/L KH_2_PO_4_). When required, antibiotics were added to the growth media at the following final concentrations: spectinomycin, 100 µg/mL; chloramphenicol, 50 µg/mL for both *E. coli* and *Z. mobilis*. All *Z. mobilis* and derivative strains used in this study are listed in Table 1.

**Table 1:** List of *Z. mobilis* strains used in this study

Strains	Description	Note
*Z. mobilis* ZM4	*Z. mobilis* subsp. *mobilis* ZM4 (wild-type strain)	ATCC31821
ZM-Cas12a	The Cas12a expressing cassette with spectinomycin resistance integrated into *ZMO0038* locus	This study
ZM-Δ0028	ZM-Cas12a with deletion of *ZMO0028*	This study
ZM-ΔHfq	ZM-Cas12a with deletion of *ZMO0347*	This study
ZM-mCherry	*ZMO0028* replaced by the reporter gene *mCherry*	This study
ZM-LdhBc	*ZMO0028* replaced by gene *LdhBc*	This study
ZM-Cas12a-ΔpZM33	ZM-Cas12a with native plasmid pZM33 cured	This study
ZM-Cas12a-ΔpZM36	ZM-Cas12a with native plasmid pZM36 cured	This study
ZM-Cas12a-ΔpZM39	ZM-Cas12a with native plasmid pZM39 cured	This study

### DNA manipulation techniques

All constructs used in the study are listed in Additional file 2: Table S1. Sequences of the primers, crRNAs, and oligonucleotides used in the study are listed in Additional file 3: Table S2. Plasmids and chromosomal DNA were extracted using AxyPrep kits (Corning, China). DNA polymerases used were PrimerSTAR (Takara, Japan) or Taq DNA polymerases (Tsingke, China). Restriction endonucleases and T4 DNA ligase were from Thermo Scientific (USA), and the isothermal assembly method was used in this work [35]. Gene deletions were confirmed by PCR and Sanger sequencing (Tsingke, China).

### Generation of Cas12a-Targeting gRNA constructs

Plasmid pEZ-sgr was used as the source for crRNA guides in *Z. mobilis*. This plasmid is a derivative of pEZ15A that carries a minimal CRISPR array containing synthetic promoter PJ23119 and two *Bsa*I restriction sites for easy cloning of new spacers. The synthetic promoter PJ23119 was used to transcribe target-specific sgRNA. The Cas12a-targeting gRNA sequence was annealed using two single-stranded oligonucleotides and ligated into *Bsa*I-linearized pEZ-sgr.

Since the PAM for Cas12a were reported to be 5′-TTN [34], and PAM GTTC and TTTN yielded stronger repression in comparison to a non-targeting RNA control [37]. Collectively, the PAM sequence 5′-TTTN-3′was used in this study. Oligonucleotides for spacers were designed as follow: the coding region of the target genes were screened for the presence of a PAM of 5′-TTTN-3′. For every PAM found, 23-nt downstream sequence was selected as the potential target sequence, then oligonucleotides were designed as 5′-AGAT + (target sequence)-3′ and 5′-TGAC + (reverse complement of the target sequence)-3′. A list of all spacers tested in this study is provided in Additional file 2: Table S2. In addition, the homologous regions flanking the editing sites were also included as a donor for homology dependent repair purposes. The customized specific plasmid was then transferred into Cas12a-expressing *Z. mobilis* strain ZM-Cas12a.

### Electroporation of *Z. mobilis*

Electro-competent *Z. mobilis* was prepared as described before with slight modifications [48]. Briefly, a single colony was inoculated into 5-mL RMG5 media and grown without shaking at 30 °C for 24 h as the seed culture. The seed culture was then transferred into the screw-cap bottle. Cell culture was placed on ice for 30 min and cells were collected by centrifuging when reached an OD_600_ value of 0.4–0.6. Cell pellets were washed once with ice-cold sterile water, re-centrifuged, and washed twice with pre-chilled sterilized 10% (v/v) glycerol. These pellets were resuspended in 10% glycerol at a concentration approximately 1000 folds higher than the starting culture. Competent cells were stored at − 80 °C as small aliquots.

*Z. mobilis* cells were transformed with plasmids by electroporation (Bio-Rad Gene Pulser, 0.1-cm gap cuvettes, 1.6 kV, 200 Ω, 25 μF). After electroporation, 1-mL RM medium was added to the electroporation mixture and cells were recovered at 30 °C for 3–5 h. The revived culture was plated on solid mating media containing appropriate antibiotics, and then incubated at 30 °C for 2–3 days for transformation efficiency determination.

### Curing of targeting plasmids

In order to cure the targeting plasmid, the transformant harboring the targeting plasmid after genome editing was inoculated into RMG5 medium without antibiotics selection pressure for 8 to 16 h, which were then spread on RMG5 plates without antibiotic. Colonies were confirmed as targeting plasmid cured by determining their sensitivity to chloramphenicol.

### Identification of edited genes

To identify positive clones with nucleotide substitutions of the *ldh* (*ZMO1237*) open reading frame, colony PCR was conducted using primers Ldh-check-F/R. PCR products were digested with *Pst*I. To confirm the deletion (or the replacement) of native gene, colonies with *ZMO0028* deletion or replacement of either *LdhBc* or *mCherry* were identified by colony PCR using primers 0028check-F/R. Colonies with *ZMO0347* (*Hfq*) deletion were identified by PCR primers 0347-check-F/R. Colonies with correct PCR product sizes were selected as candidates and confirmed by Sanger sequencing (Tsingke, China).

### Fluorescence measurements

The protocol used for flow cytometry analysis of fluorescence was modified slightly from a previous study [41, 49]. Briefly, cells were washed with phosphate buffered saline (PBS) twice and then resuspended into PBS to a concentration of 10^7^ cells/mL. Cells were analyzed by flow cytometry using Beckman CytoFLEX FCM (Beckman Coulter, USA) with the PBS as the sheath fluid. The fluorescence of mCherry was excited with the 561 nm and detected with PC5.5 [50–52]. To avoid rare events which could affect the population distribution, at least 20,000 events of each sample were analyzed. Data were processed via FlowJo software (FlowJo, LLC, USA).

### Construction of a lactate producing strain, and the cultivation assay

To construct a lactate producing strain, a heterologous lactate dehydrogenase from *Bacillus coagulans* (*LdhBc*) was codon optimized and synthesized. The native promoter *PadhB* driving the alcohol dehydrogenase (ZMO1596) involved in ethanol production was used to stimulate the expression of *LdhBc*. The *LdhBc* flanked 800 bp upstream and downstream of *ZMO0028* was cloned into the pEZ-sgr vector to generate the donor plasmid. A specific crRNA targeting *ZMO0028* was designed and assembled to the donor plasmid. The resulted plasmid was induced to the ZM4-Cas12a through Cas12a-mediated genome editing. Recombinant strain was identified by PCR and confirmed by Sanger sequencing. The correct strain was named ZM-LdhBc.

The seed culture of ZM4 and lactate producing strain ZM-LdhBc were firstly revived from frozen glycerol stocks in RM at 30 °C for 6 ~ 8 h without shaking. Then seed culture were transferred into 100-mL shake flasks containing 80 mL RM medium at an initial OD_600_ ≈ 0.08. Cultures were inoculated at 30 °C with a shaking speed of 100 rpm. Three replicates were used for each strain. Samples from the shake flasks were taken at various time points and the biomass was evaluated by determination of optical density (OD_600 nm_) using spectrometer. Culture supernatants were collected and filtered through a 0.22 μm filter for further analysis.

### High-pressure liquid chromatography (HPLC) analysis

High-pressure liquid chromatography (HPLC) was performed using a Shimadzu HPLC system (Japan) equipped with Aminex Resin-Based Columns (Bio-Rad) and refractive index detector (RID) to quantify glucose, ethanol, and lactate. The mobile phase is 5 mM H_2_SO_4_, and the flow rate was set as 0.5 mL/min at 60 °C.

## Supplementary information

**Additional file 1: Figure S1.** The transformation efficiency of *Z. mobilis* ZM4 and the Cas12a expressing strain with or without the expression of crRNA targets. ZM4 represented the wild-type strain *Zymomonas mobilis* ZM4, ZM4-Cas12a represented the recombinant strain with Cas12a integrated at the ZMO0038 locus of ZM4 genome. pEZ-sgr and its derivates containing crRNA scaffold, the pEZ-Cas12a expressing both the Cas12a effector nuclease and the crRNA via a single plasmid. Values are the means of experiments with three or more technical replicates; error bars are standard deviation.

**Additional file 2: Table S1.** List of plasmids used in this study.

**Additional file 3: Table S2.** Primers used in this study.

## Data Availability

The authors declare that all the data supporting the findings of this study are available within the paper and its Supplementary Information files or are available from the corresponding author on request.

## Abbreviations

Cas: CRISPR associated protein
CRISPR: Clustered Regularly Interspaced Short Palindromic Repeats
crRNA: CRISPR RNA
DR: direct repeat
DSB: double-strand break
ED: Entner–Doudoroff pathway
FCM: flow cytometry
HPLC: high-pressure liquid chromatography
HR: homologous recombination
Ldh: lactate dehydrogenase
NHEJ: nonhomologous end-joining
PAM: protospacer adjacent motif
PDC: pyruvate decarboxylase
RID: Refractive index detector
sgRNA: single-strand guide RNA
ssDNA: single-stranded DNA
