# Supplementary material for: Establishment and application of a CRISPR–Cas12a assisted genome-editing system in Zymomonas mobilis
Source: Microb Cell Fact. 2019 Oct 3;18:162. doi: 10.1186/s12934-019-1219-5 (PMC6777028; doi:10.1186/s12934-019-1219-5)
Supplement: Supplementary file 1 — Additional file 1: Figure S1. The transformation efficiency of Z. mobilis ZM4 and the Cas12a expressing strain with or without the expression of crRNA targets. ZM4 represented the wild-type strain Zymomonas mobilis ZM4, ZM4-Cas12a represented the recombinant strain with Cas12a integrated at the ZMO0038 locus of ZM4 genome. pEZ-sgr and its derivates containing crRNA scaffold, the pEZ-Cas12a expressing both the Cas12a effector nuclease and the crRNA via a single plasmid. Values are the means of experiments with three or more technical replicates; error bars are standard deviation. [file 12934_2019_1219_MOESM1_ESM.docx]

**Figure S1.** The transformation efficiency of *Z. mobilis* ZM4 and the Cas12a expressing strain with or without the expression of crRNA targets. ZM4 represented the wild-type strain *Zymomonas mobilis* ZM4, ZM4-Cas12a represented the recombinant strain with Cas12a integrated at the ZMO0038 locus of ZM4 genome. pEZ-sgr and its derivates containing crRNA scaffold, the pEZ-Cas12a expressing both the Cas12a effector nuclease and the crRNA via a single plasmid. Values are the means of experiments with three or more technical replicates; error bars are standard deviation.
